# LRRK2 is involved in the pathogenesis of system lupus erythematosus through promoting pathogenic antibody production

**DOI:** 10.1186/s12967-019-1786-6

**Published:** 2019-01-22

**Authors:** Meiyu Zhang, Chengcheng Yao, Jun Cai, Shuai Liu, Xia-nan Liu, Yingying Chen, Shujun Wang, Ping Ji, Meng Pan, Zizhen Kang, Ying Wang

**Affiliations:** 1 Shanghai Institute of Immunology, Department of Immunology and Microbiology, Shanghai Jiao Tong University School of Medicine, Shanghai, 200025 China; 2 Department of Dermatology, Rui Jin Hospital, Shanghai Jiao Tong University School of Medicine, Shanghai, 200025 China; 3 Department of Inflammation and Immunity, Cleveland Clinic, Cleveland, OH 44195 USA; 4 Department of Molecular Medicine, Cleveland Clinic Lerner College of Medicine, Case Western Reserve University, Cleveland, OH 44106 USA; 5 Department of Clinical Laboratory, The First Affiliated Hospital of Zhengzhou University, Zhengzhou, 450052 China; 6 Department of Pathology, Shanghai Jiao Tong University School of Medicine, Shanghai, 200025 China

**Keywords:** System lupus erythematosus, Leucine-rich repeat kinase 2, Lupus-like mouse model, B cell differentiation, Antibody production

## Abstract

**Background:**

Systemic lupus erythematosus (SLE) is a prototypic autoimmune disease characterized by the presence of pathogenic autoantibodies associated with polyclonal B cell hyperreactivity. Previous study reported that autophagy-related gene Leucine-rich repeat kinase 2 (LRRK2) was likely a susceptible gene for SLE. However, the pathogenic function of LRRK2 in SLE is undefined.

**Methods:**

Using quantitative PCR, we compared the expression levels of LRRK2 in B cells between SLE patients and healthy controls. The expression levels of *LRRK2* in in vitro induced CD19^hi^ B cells and naïve B cells were compared as well based on RNA-seq assay. A pristane-induced lupus-like mouse model was used to explore the effects of LRRK2 on the development of SLE. IgG level, B cell subsets in the spleens and bone marrows and pathological features in the kidneys were compared between wildtype (WT) and *Lrrk2*^−*/*−^ littermates.

**Results:**

It was obvious that *LRRK2* expression was dramatically up-regulated in primary B cells from SLE patients compared to those from healthy controls, as well as in activated CD19^hi^ B cells. More significantly, *LRRK2* expression in B cells was positively correlated with system lupus erythematosus disease activity index (SLEDAI), an indicator for disease severity, and serum IgG levels in SLE patients. Negative correlations were observed between *LRRK2* expression and serum C3 or C4 levels, two clinical features associated with SLE-related nephritis. *LRRK2* deficiency reduced the death rate of pristane treated mice. Decreased levels of total IgG and autoantibody were detected in the serum with less deposition of immune complexes and attenuated pathological symptoms in the kidneys of *Lrrk2*^−*/*−^ mice. Consistent with the reduction in IgG production, the percentages of germinal center B cells and plasma cells decreased significantly as well with LRRK2 deficiency.

**Conclusions:**

Our study demonstrates that LRRK2 expression is upregulated in B cells from SLE patients with strong correlations to disease severity. LRRK2 deficiency largely attenuates the pathogenic progress of lupus-like features in pristane-induced mice. This is probably achieved through affecting B cell terminal differentiation and subsequent immunoglobulin production.

**Electronic supplementary material:**

The online version of this article (10.1186/s12967-019-1786-6) contains supplementary material, which is available to authorized users.

## Background

Systemic lupus erythematosus (SLE) is a severe heterogeneous systemic autoimmune disease characterized by the production of autoantibodies against certain self-antigens. The formation and deposition of immune complexes (ICs) in various tissues and organs result in local inflammatory responses and severe tissue destruction including the brain, blood, heart and kidney [1]. Polyclonal B cell hyperreactivity is considered to be one of the main immuno-pathological properties in SLE [2]. Successful application of Rituximab, a chimeric mouse-human monoclonal antibody against B cell-specific antigen CD20, in clinic highlights the role of B cells in the pathogenesis of SLE [3, 4].

It is demonstrated that B cell activation in SLE is comprehensively regulated by both intrinsically genetic and extrinsically environmental factors [5]. Multiple cytokines related to B cell activation, such as IFN-α mainly derived from plasmacytoid dendritic cells (pDCs) and Th2 cytokines such as IL-4, IL-5 et al., are elevated in SLE patients [6, 7]. Most intrinsic factors are implied from the viewpoint of genetics based on genome-wide associated analysis (GWAS) [8]. Among them, an autophagy-related gene encoding leucine-rich repeat kinase 2 (LRRK2) was recently identified [9]. LRRK2 contains 2527 amino acid residues with several functional domains, including a leucine-rich repeats (LRR) domain, a Ras of complex proteins (ROC) domain, a C-terminal of ROC (COR) domain, a kinase domain and a WD40-repeat domain [10]. LRRK2 is reported to be highly expressed in certain immune cells, such as B cells, monocytes, macrophages, and microglia whereas less in T cells [11]. It has recently been reported to be associated with multiple diseases including Parkinson’s disease [12, 13], leprosy [14], inflammatory bowel disease (IBD) [15, 16] and cancers [17].

Emerging data suggest that LRRK2 plays critical roles in immune modulation of macrophages, which is supported by the investigations on the resistance of LRRK2 to intracellular pathogen infection, including *Listeria monocytogenes* [18] and *Mycobacterial tuberculosis* [19]. Our recent study also suggested that LRRK2 was critical for NLRC4 inflammasome activation in macrophages, which was indispensable for host defense against Salmonella infection [20]. In addition to the involvement of LRRK2 in innate immunity, the roles of LRRK2 in B cells were firstly proposed due to its high expression in peripheral B cells in an age-dependent manner [21]. Considering its susceptibility to SLE and high expression in B cells, whether LRRK2 functions in the pathogenesis of SLE is worthy of investigation.

In this study, we found that LRRK2 expression was dramatically increased in B cells from SLE patients compared to that from healthy controls (HCs). Of note, LRRK2 expression in B cells was positively correlated with disease severity and the levels of serum IgG in SLE patients. Furthermore, we demonstrated that LRRK2 promoted B cell terminal differentiation, humoral immune response and consequently lupus-like syndrome in a pristane-induced mouse model, thus implicating LRRK2 as a novel target in SLE therapy.

## Methods

### Human subjects

SLE patients (n = 22) enrolled in this study were from Ruijin Hospital affiliated to Shanghai Jiao Tong University School of Medicine (Shanghai, China). All SLE patients fulfilled the American Rheumatism Association Criteria for the diagnosis of SLE. The study was approved by the Ethic Committee of Ruijin Hospital affiliated to Shanghai Jiao Tong University School of Medicine. All experiments were performed according to the principles of the Declaration of Helsinki. Informed consent forms were assigned individually. HCs (n = 31) were volunteer donors undergoing annual physical examination. SLE patients and HCs were both gender and age matched (Table 1).

**Table 1:** Demographic and clinical characteristics of SLE and healthy controls

Characteristics	Healthy controls (n = 31)	SLE patients (n = 22)
Age (years)	42.29 ± 11.38 (24–62)	36.29 ± 13.79 (13–71)
Gender (female/male)	27/4	20/2
Disease duration (years)	–	6.5 ± 6.07
Renal diseases n (%)	–	15 (68.1)
Neurological disorder n (%)	–	3 (13.6)
Thrombocytopenia n (%)	–	5 (22.7)
Anti-ds-DNA antibody n (%)	–	15 (68.1)
IgG (mg/dL)	–	1277 ± 388
IgM (mg/dL)	–	81 ± 59
IgA (mg/dL)	–	300 ± 128
SLEDAI	–	6.27 ± 5.22 (2–20)

### Cell isolation

Whole blood was collected in heparin lithium-treated tubes. Peripheral blood mononuclear cells (PBMCs) were isolated by density gradient centrifugation using Lymphoprep™ reagent (Axis-shield, Norway). CD4^+^ T cells from SLE patients or HCs were isolated by using a human CD4 MicroBeads kit (Miltenyi Biotec, Bergisch Gladbach, Germany) according to the manufacturer’s instructions. Briefly, PBMCs were incubated in PBS supplemented with 0.5% bovine serum albumin (BSA) containing CD4 MicroBeads. Cell suspension was applied on an MS separation column placing in the magnetic field. Unlabeled cells were collected for subsequent B cell purification. The MS column was removed from the magnetic field and CD4^+^ T cells were eluted by rinsing the column with PBS. B cells were further purified with an EasySep™ Human Naïve B Cell Enrichment Kit (STEMCELL Technologies, Canada) accordingly. Briefly, the elution cell fraction after CD4^+^ T cell purification was incubated with enrichment cocktail for 10 min at room temperature (RT). Magnetic particles were added in the mixture for 5 min incubation at RT. The tubes were placed in the magnet at RT for 5 min. The enriched cell suspension was then carefully transferred into a new tube. The purity of CD4^+^ T cells and B cells was determined by flow cytometry. Cells with the purity > 95% were used for further experiments.

### RNA extraction, reverse transcription, and quantitative PCR

Total RNA was extracted from PBMCs, purified CD4^+^ T cells or B cells by using the TRIzol reagent (Invitrogen, Carlsbad, CA, USA). NanoDrop spectrophotometer (Thermo Scientific, Waltham, MA, USA) was applied for RNA concentration determination. cDNA was synthesized by using the PrimeScriptTM RT reagent Kit (TaKaRa, Kusatsu, Japan). Semi-quantitative polymerase chain reaction (qPCR) was carried out with the SYBR PremixEx TaqTM II kit (TaKaRa) in a ViiA 7 Real-Time PCR System (Applied Biosystems, Carlsbad, CA, USA). The primers specific to *LRRK2* were as forward: 5′-GAGCACGCCTCCAAGTTATTT-3′ and reverse: 5′-ACTGGCATTATGAACTGTTAGCA-3′. House-keeping gene *GAPDH* was used as an internal control (primers: forward: 5′-GGAGCGAGATCCCTCCAAAAT-3′; reverse: 5′-GGCTGTTGTCATACTTCTCATGG-3′). The expression level of *LRRK2* was calculated based on cycle threshold (Ct) values of target gene and *GAPDH*. The relative expression level of *LRRK2 *= 2^(Ct_*GAPDH*_ − Ct_*LRRK2*_).

### Mice

*Lrrk2*^−*/*−^ mice (C57BL/6 N-*Lrrk2*^*tm1.Mjff/J*^; approved by the Michael J. Fox Foundation for Parkinson’s research; JAX stock 016121) were purchased from the Jackson Laboratory (USA) and backcrossed onto a C57BL/6 background for at least six generations. All mice were bred and maintained in individually ventilated cages under specific pathogen-free conditions in the accredited animal facility of Shanghai Jiao Tong University School of Medicine (Shanghai, China). Age- and gender-matched wildtype (WT) mice at 8–10 weeks’ old were used as littermate controls in all experiments. To ameliorate the suffering of mice, all mice were sacrificed through CO_2_ inhalation in the studies.

### Preparation of lupus-like mice by pristane injection

8–10-week-old age- and gender-matched WT and *Lrrk2*^−/−^ female mice were administered intraperitoneally with 500 μL pristane (2, 6, 10, 14-tetramethylpentadecane) (Sigma-Aldrich, St Louis, MO, USA). 50–100 μL blood was collected from eye veins with an interval of 1 month for serum preparation. Mice were sacrificed at 9 months after pristane injection. Immunological and pathological characters were assessed.

### Flow cytometry

The spleens were collected from WT and *Lrrk2*^−/−^ pristane-treated mice at 9 months. Splenocytes were prepared by density gradient centrifugation using Lymphoprep™ reagent (Axis-shield). For flow cytometry, splenocytes were washed once with FACS buffer (1× PBS supplemented with 2% fetal bovine serum) (Millipore, Billerica, MA, USA) and stained with antibodies against surface molecules, including anti-CD45R/B220-Percp-cy5.5, anti-CD93-phycoerythrin/cyanin (PE-Cy7), anti-IgM-allophyco-cyanin (APC-Cy7), anti-CD38-APC, anti-CD138-APC, anti-GL7-fluorescein isothiocyanate (FITC) (all from Biolegend, San Diego, CA, USA) and anti-CD23-PE (Thermo Scientific) antibodies diluted in FACS buffer. Cells were incubated at 4 °C for 30 min, and collected on an LSR Fortessa (BD Biosciences, San Diego, CA, USA). Data analysis was carried out with FlowJo 7.6 software (Tree Star, Ashland, OR, USA).

### Enzyme linked immune sorbent assay (ELISA)

Blood was kept at RT for 2 h and centrifuged at 5500 rpm for 10 min. Sera were collected and stored at − 80 °C for ELISA assay. For determination of total IgG and IgM in the serum, 96-well polystyrene plates (Corning, NY, USA) were coated with 2 µg/mL goat anti-mouse IgG(H + L) or goat anti-mouse IgM (SouthernBiotech, Birmingham, AL, USA) antibodies diluted in bicarbonate/carbonate coating buffer (15 mM Na_2_CO_3_ and 35 mM NaHCO_3_, pH 9.6), and incubated at 4 °C overnight. After washing 3 times with PBST (PBS containing 0.05% Tween-20) (Sangon Biotech, Shanghai, China), the wells were blocked with 200 µL/well PBS containing 5% skimmed milk powder at RT for 2 h. Mouse sera were serially diluted in PBST containing 3% skimmed milk powder. 100 µL diluted serum was added to each well and the plates were incubated at RT for 2 h. After washing 3 times with PBST, the wells were incubated with horseradish peroxidase (HRP)-conjugated goat anti-mouse antibody for IgG(H + L) and HRP-conjugated goat anti-mouse antibody for IgM (SouthernBiotech) working solution (1:4000 diluted in PBST containing 3% skimmed milk powder, 100 µL/well) at RT for 2 h. Tetramethylbenzidine (TMB) (BD Bioscience) solution (100 µL/well) was added, and the plates were incubated at RT in the dark for 15 min. The reaction was stopped by adding 1 M H_2_SO_4_ (50 µL/well). The absorbance at 450 nm was detected within 5 min by PowerWaveXS2 microplate spectrophotometer (BioTek, Burlington, VT, USA).

For the detection of anti-ribonucleoprotein (RNP) and anti-double strand DNA (dsDNA) antibodies, 96-well ELISA plates (Corning) were coated with 0.3 µg/mL (100 µL/well) U1-SnRNP (for anti-RNP autoantibody) (Diarect, Freiburg, Germany) and 3 µg/mL (100 µL/well) dsDNA (for anti-dsDNA Ab) (Diarect) diluted in coating buffer and incubated at 4 °C overnight. After washing 3 times with PBST, the wells were blocked with 200 µL/well blocking buffer at RT for 2 h. 100 µL/well diluted serum was added to the wells and the plates were incubated at RT for 2 h. After washing 3 times with PBST, HRP-conjugated goat anti-mouse antibody for IgG(H + L) (SouthernBiotech) working solution (1:4000 diluted in PBST containing 3% skimmed milk powder, 100 µL/well) was added and incubated at RT for 2 h. TMB (BD Bioscience) solution (100 µL/well) was added later, and the plates were incubated at RT in the dark for 15 min. The reaction was stopped by adding 1 M H_2_SO_4_ (50 µL/well). The absorbance at 450 nm was detected within 5 min by PowerWaveXS2 microplate spectrophotometer (BioTek).

### Histology and immunofluorescence assay (IFA)

Mouse kidneys were fixed in PBS containing 4% paraformaldehyde at RT and embedded in the paraffin routinely. Paraffin sections (4 μm) were stained with hematoxylin and eosin (H&E). Sections were scanned with OLYMPUS microscope (Tokyo, Japan) equipped with a 40× objective. Glomerulonephritis in the kidneys was assessed using a semiquantitative (1–4+) scoring scale based on the number of the glomeruli with endocapillary proliferation and extracapillary proliferation (crescents) as following: no glomeruli = 0; 1–25% of glomeruli = 1; 26–50% of glomeruli = 2; 51–75% of glomeruli = 3; and 76–100% of glomeruli = 4+. The slides were double-blinded examined by a pathologist. At least five randomly selected images were undergone analysis.

The deposition of IgG in the kidneys was examined by direct IFA with paraffin sections. The slides were stained with fluorochrome-conjugated anti-IgG Ab (Abcam, #ab6785) with a 1:500 dilution and incubated at 4 °C for 24 h. The slides were washed with PBS and air-dried. 4,6-diamidino-2-phenylindole (DAPI) Fluoromount-G (YEASEN, Shanghai, China) was added on the slides before the observation. Fluorescence signal was determined under a TCS SP8 confocal microscope (Leica, Solms, Germany). Data analysis was carried out with ImageJ software (National Institutes of Health, Bethesda, MD, USA). At least five fields were randomly selected for quantification analysis.

### Statistical analysis

Data were presented as mean ± standard error of means (S.E.M). Statistical analyses were performed by using Graphpad Prism 5.0 software (Graphpad Prism, La Jolla, CA, USA). Statistic difference was determined by unpaired *Student t* test for the data with gaussian distribution, and by Mann–Whitney test for those with non-gaussian distribution. Unless stated, p < 0.05 was considered statistically significant.

## Results

### Up-regulation of LRRK2 in B cells from SLE patients as well as in activated B cells

**Fig. 1:**
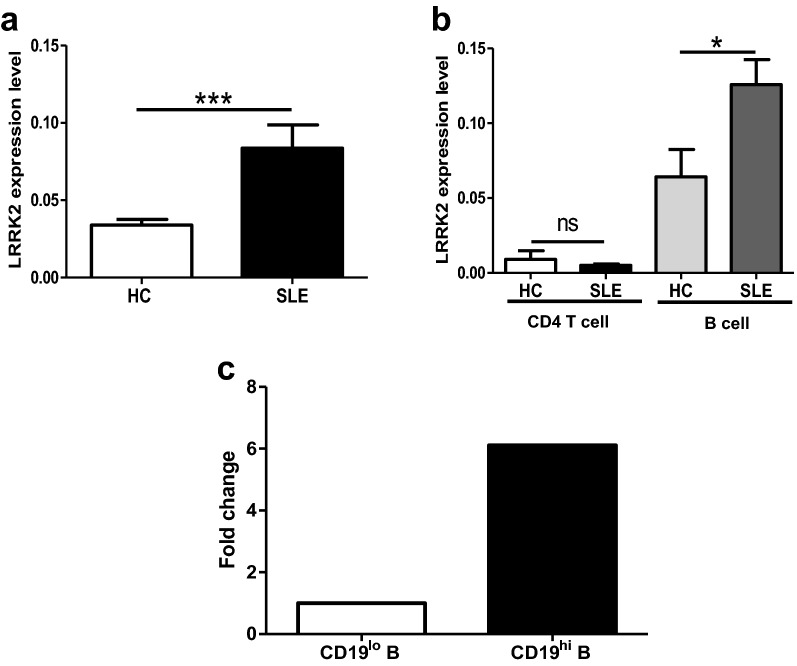
LRRK2 expression profiles in the peripheral immune cells. Whole blood was collected from SLE patients and healthy volunteer donors. The expression levels of *LRRK2* in PBMCs (**a**), CD4^+^ T cells and B cells (**b**) from SLE patients and healthy donors were determined by RT-qPCR. **c** The expression levels of *LRRK2* in resting CD19^lo^ B cells and CD19^hi^ B cells from three healthy donors were calculated based on transcriptome assay data [24]. *p < 0.05; ***p  <  0.001

To explore the possible relationship between LRRK2 and SLE pathogenesis, the expression levels of LRRK2 in PBMCs were firstly compared between SLE patients and HC donors (Table 1). Consistent with the previous study [9], *LRRK2* expression in PBMCs was significantly increased in SLE patients when compared to HCs (Fig. 1a). CD4^+^ T cells and B cells from SLE patients or HCs were further isolated separately with high purity (Additional file 1: Figure S1) and the expression levels of *LRRK2* in cell subsets were determined. When precisely comparing *LRRK2* expression in CD4^+^ T cells and B cells, it was obvious that B cells from both SLE and HCs groups expressed *LRRK2* more dramatically than CD4^+^ T cells. More significantly, the expression of *LRRK2* was elevated in B cells from SLE patients than that from HCs whereas was comparable in CD4^+^ T cells from SLE patients and HC individuals (Fig. 1b).

CD19^hi^ B cells were previously reported existing in certain antibody-driven autoimmune diseases, such as common variable immunodeficiency (CVID) [22], SLE [23] and pemphigus [24]. These CD19^hi^ B cells exhibit activated phenotypes, and function in promoting antibody production in autoimmune diseases. CD19^hi^ B cells can be induced through co-culturing activated CD4^+^ T cells with homologous resting B cells (CD19^lo^ B cells) in vitro from healthy donors [24]. Relying on transcriptome analysis of CD19^hi^ B cells and homologous CD19^lo^ B cells from healthy donors, we compared *LRRK2* expression between these two types of B cell populations. It was found that more *LRRK2* expression was detectable in CD19^hi^ B cells than in the resting counterparts (Fig. 1c). These results strongly imply that LRRK2 expression is associated with B cell activation and antibody production, which might be involved in the pathogenesis of SLE.

### Upregulation of LRRK2 in B cells is associated with disease severity of SLE

**Fig. 2:**
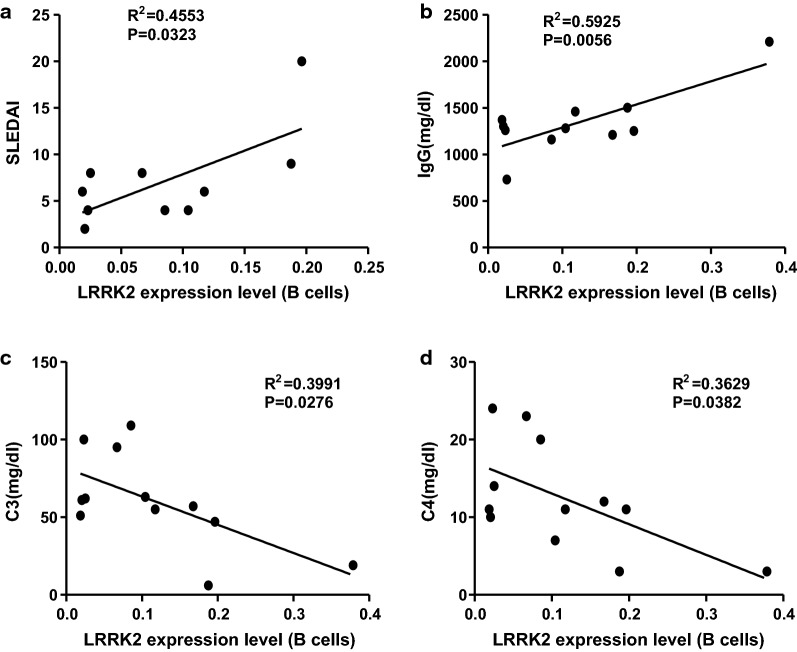
Correlation analysis between LRRK2 expression in B cells and clinical manifestations of SLE. *LRRK2* expression in purified B cells from SLE patients was determined by semi-qPCR with *GAPDH* as an internal control. Correlations between *LRRK2* expression in B cells and SLEDAI (**a**), IgG level (**b**), C3 (**c**) and C4 (**d**) were determined. The correlation coefficient r and the p value were calculated using the Spearman rank test

To further support the possibility of LRRK2 involvement in SLE, we investigated the correlations between LRRK2 expression and key clinical manifestations related to disease severity of SLE that were routinely examined in clinical laboratory. A significantly positive correlation was apparent between *LRRK2* expression in B cells and system lupus erythematosus disease activity index (SLEDAI), an indicator for disease severity (Fig. 2a). Correlations between total IgG as well as autoantibody levels and LRRK2 expression in B cells were analyzed as well. It was obvious that there was a significantly positive correlation between *LRRK2* expression in B cells and serum IgG levels (Fig. 2b) whereas no strong correlation was present between *LRRK2* expression in B cells and autoantibody levels (data not shown). It is well recognized that the decrease in C3 and C4 in the periphery is associated with disease severity of SLE, especially the occurrence of SLE-related nephritis [25]. Contrarily, *LRRK2* expression in B cells was negatively correlated with both serum C3 (Fig. 2c) and C4 levels (Fig. 2d). Although not all 22 SLE patients were included in correlation study due to the incomplete clinical examination parameters, the fact that LRRK2 expression in B cells exhibits strong correlations with disease severity of SLE in partial patients implies overwhelming roles of LRRK2 in the pathologies of SLE.

### Comparable B cell subsets and serum immunoglobulin levels between WT and *Lrrk2*^−*/*−^ mice

**Fig. 3:**
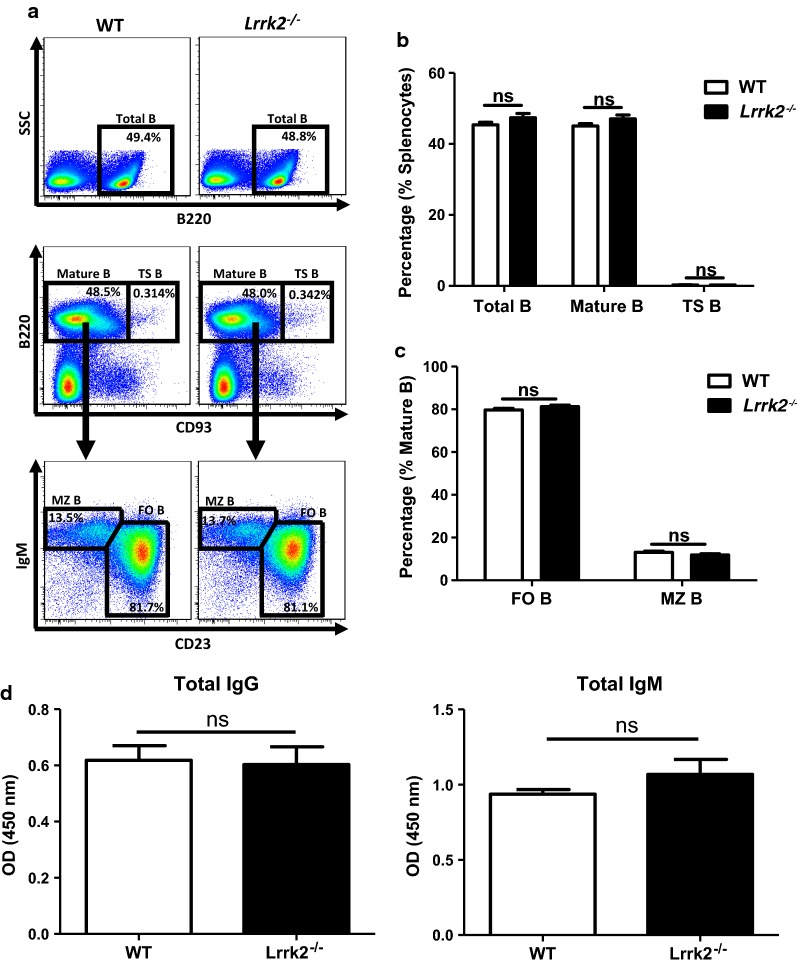
Comparable B cell subsets and serum immunoglobulin levels between WT and *Lrrk2*^−*/*−^ Mice. **a** Splenocytes were collected from WT and *Lrrk2*^−*/*−^ mice. B cell subsets in the periphery were analyzed by flow cytometry, including total B cells (B220^+^ lived cells, upper), mature B cells (B220^+^CD93^−^, middle), transition (TS) B cells (B220^+^CD93^+^, middle), marginal zone (MZ) B cells (B220^+^CD93^−^CD23^−^IgM^+^, low), and follicular (FO) B cells (B220^+^CD93^−^CD23^+^ IgM^low/−^, low). **b** Statistic percentages of total B cells, mature B cells and TS B cells in the spleens of WT and *Lrrk2*^−*/*−^ mice. **c** Statistic percentages of MZ and FO B cells in the spleens of WT and *Lrrk2*^−*/*−^ mice. **d** Sera from WT and *Lrrk2*^−*/*−^ mice were subjected to the determination of total IgG and IgM by ELISA as described in “Methods”. OD_450_ value was detected to represent IgG (left) and IgM (right) levels in the serum of the mice. Each group had at least six mice for the analysis. *ns* not significant

To define the role of LRRK2 in SLE development, *Lrrk2*^−*/*−^ mice were introduced in our study. With abrogated LRRK2 expression in *Lrrk2*^−*/*−^ mice (Additional file 1: Figure S2), the effects of LRRK2 on humoral immunity at a steady state were determined first. There was no significant difference in the cellularity of total splenocytes between WT and *Lrrk2*^−*/*−^ mice (data not shown). We further examined B cell subsets in the spleens of WT and *Lrrk2*^−*/*−^ mice. After leaving bone marrow, immature B cells embark the spleens and differentiate into mature B cells through a transition stage [26]. Comparable percentages of transition (TS) B cells (B220^+^CD93^+^) were present in the spleens of WT and *Lrrk2*^−*/*−^ mice. Disruption of *Lrrk2* gene had no effect on the development of mature B cells (B220^+^CD93^−^) and follicular (FO) B cells (B220^+^CD93^−^CD23^+^IgM^low/−^), two subpopulations of peripheral B cells either (Fig. 3a–c). A fraction of bone marrow B cell subset migrates to the marginal zone of the spleens constituting marginal zone (MZ) B cells (B220^+^CD93^−^CD23^−^IgM^+^). This population represents long-lived B cells with the ability to respond to T-independent antigens from blood-borne pathogens [27]. Similarly, no difference in MZB cells was observed between WT and *Lrrk2*^−*/*−^ mice either (Fig. 3a, c). Therefore, mice lack of LRRK2 displays no impairment in B cell development.

Simultaneously, we also measured the basal levels of IgG and IgM in the serum by ELISA. It was found that IgG and IgM levels were identical between *Lrrk2*^−*/*−^ mice and WT littermates (Fig. 3d), further supporting the fact that no commitment of LRRK2 occurs during B cell development and differentiation at the steady state.

### LRRK2 deficiency reduces antibody production in pristane-treated mice

**Fig. 4:**
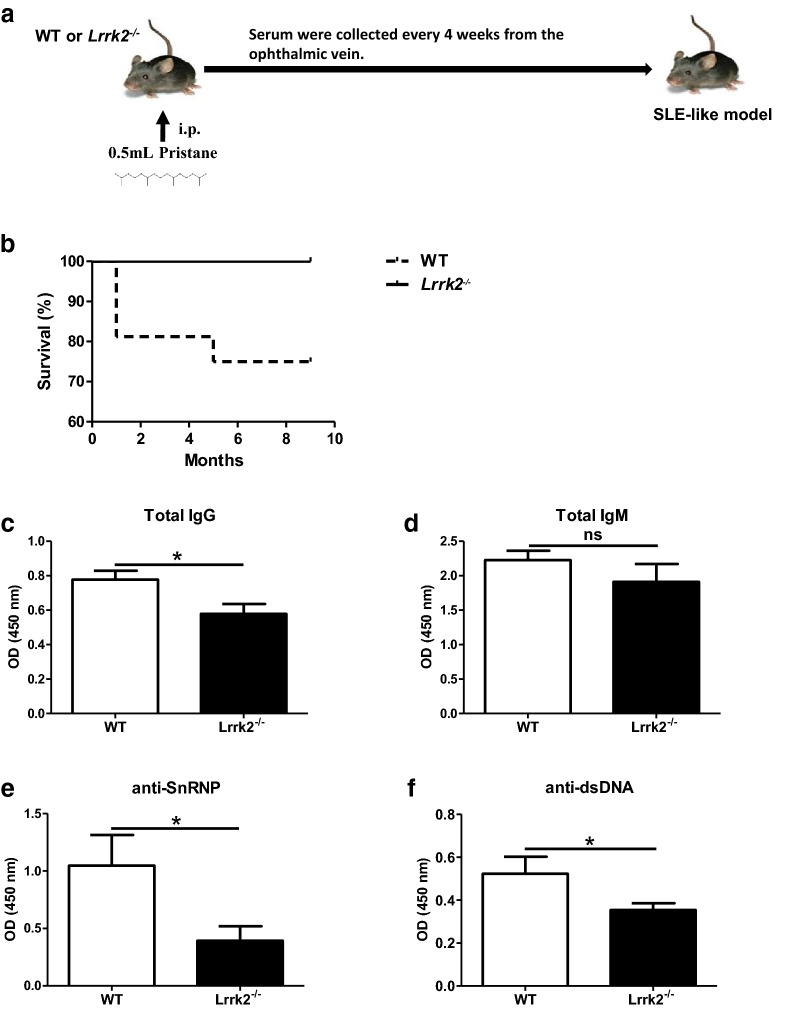
LRRK2 deficiency attenuates pristane-induced antibody production in the mice. **a** Scheme of experiment design. WT and *Lrrk2*^−*/*−^ mice were intraperitoneally injected with 0.5 mL pristane. Sera were collected monthly for IgG and IgM determination. Mice were sacrificed 9 months later. **b** Survival rate of WT and *Lrrk2*^−*/*−^ mice after pristane injection. **c**–**f** Antibody levels in the sera of WT and *Lrrk2*^−*/*−^ mice were assayed by ELISA at 9 months after pristane injection, including total IgG (**c**), total IgM (**d**), anti-SnRNP antibody (**e**) and anti-dsDNA antibody (**f**). Each group had at least six mice for antibody analysis. *p < 0.05; *ns* not significant

Pristane-induced lupus-like mouse model represents one of the models recapitulating key immunologic and clinical features of human SLE, including high percentages of IFN-α producing pDCs, production of autoantibodies against small nuclear ribonucleoproteins (snRNPs) and dsDNA, as well as the development of IC mediated glomerulonephritis [28]. Although *Lrrk2*^−*/*−^ mice exhibit normal B cell development and function at the steady state, whether pristane treatment induces different lupus-like phenotypes is worthy of investigation (Fig. 4a). It was found that there was an increased survival rate of *Lrrk2*^−*/*−^ mice in comparison to WT mice after pristane injection (Fig. 4b). We next compared the antibody production between WT and *Lrrk2*^−*/*−^ mice. The levels of serum total IgG and autoantibodies collected monthly after the injection increased gradually both in WT and *LRRK2* knockout mice (Additional file 1: Figure S3). At 9 months after pristane injection, however, total IgG level was significantly lower in *Lrrk2*^−*/*−^ mice than that in WT mice (Fig. 4c) whereas total IgM was comparable between two types of mice (Fig. 4d). When comparing autoantibody levels between WT and *Lrrk2*^−*/*−^ mice, it was obvious that both anti-snRNP and anti-dsDNA autoantibodies were lower in *Lrrk2*^−*/*−^ mice than WT counterparts where anti-snRNP was more dramatic (Fig. 4e, f).

### LRRK2 deficiency attenuates pristane-induced lupus-like pathology in mice

**Fig. 5:**
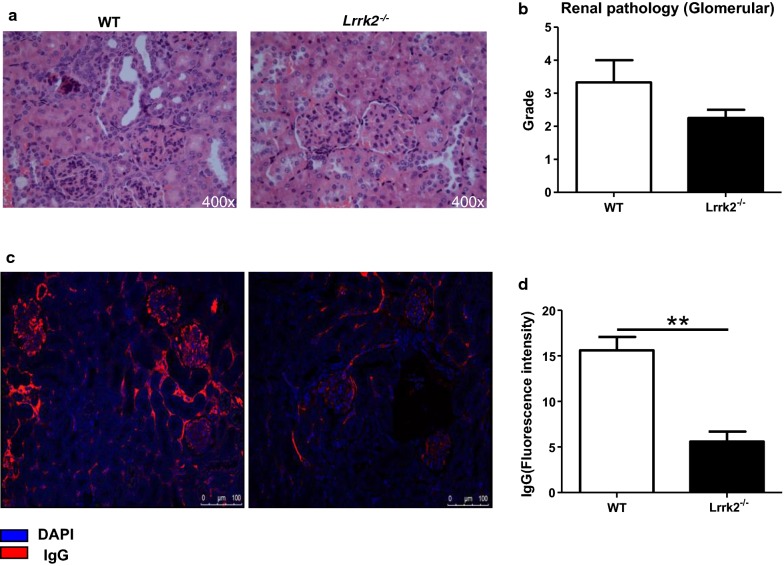
Pathological properties in mouse kidneys after pristane treatment. **a** The kidney pathology was investigated at 9 months post pristane treatment. Glomerular injury with mesangial expansion and lymphocyte infiltration in the kidneys of WT and *Lrrk2*^−*/*−^ mice was determined by H&E staining (400×). **b** Semiquantitative analysis of glomerulonephritis scores. The scores were obtained based on the number of the glomeruli with endocapillary proliferation, extracapillary proliferation (crescents), the damage grade was evaluated as described in “Methods”. **c** Representatives of glomerular IgG deposition in the kidneys of WT and *Lrrk2*^−*/*−^ mice (200×). **d** The mean fluorescence intensity (MFI) of IgG within glomeruli was determined by ImageJ software. Both statistics were based on at least five randomly selected images of each mouse. Each group had at least six mice for pathological analysis. **p < 0.01

Considering that high LRRK2 expression in B cells from SLE patients was strongly correlated with low levels of serum C3 and C4, two indicators related to renal injury during SLE pathogenesis [29], whether decreased total IgG and autoantibodies levels in *Lrrk2*^−*/*−^ mice upon pristane treatment led to less kidney pathology was further investigated at 9 months post pristane treatment. It was revealed that the severity of nephritis was ameliorated in *Lrrk2*^−*/*−^ mice reflected by less glomeruli represented by less endocapillary or extracapillary proliferative lesions and sclerotic lesions (Fig. 5a, b). The deposit of IgG in the kidneys of *Lrrk2*^−*/*−^ mice was reduced significantly as well (Fig. 5c, d). Altogether, our results indicate that LRRK2 deficiency exhibits the protection against pristane-induced lupus-like pathology, partially owing to less production of total IgG and autoantibodies.

### Decreased percentages of germinal center B cells and plasma cells in *Lrrk2*^−*/*−^ mice after pristane injection

**Fig. 6:**
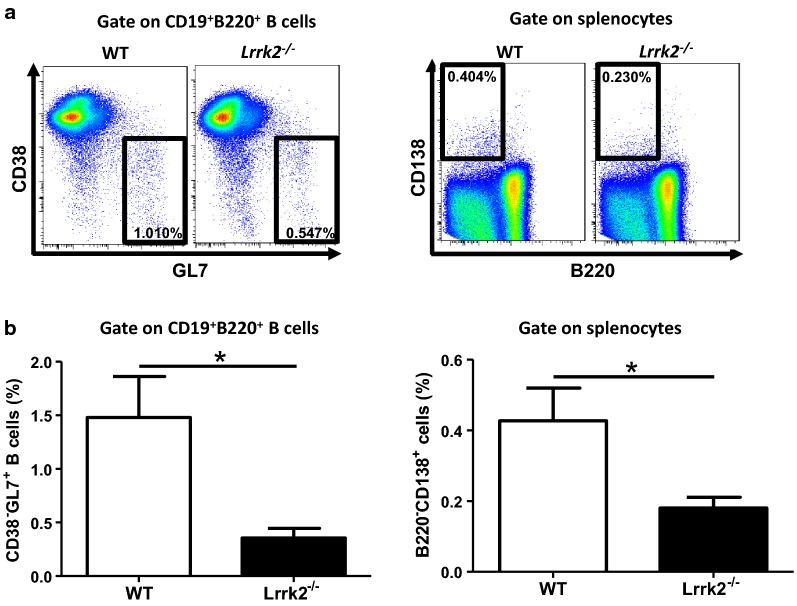
Decreased percentages of GC B cells and plasma cells in *Lrrk2*^−*/*−^ mice after pristane injection. **a** Splenocytes were collected from the spleens of WT and *Lrrk2*^−*/*−^ mice at 9 months post pristane treatment. Flow cytometric analysis were performed to determine the percentages of GC B cells (CD38^−^GL7^+^, left) in CD19^+^B220^+^ cells and plasma cells (B220^−^CD138^+^, right) in the splenocytes of WT and *Lrrk2*^−*/*−^ mice. **b** Statistic results of the percentages of GC B (left) and plasma cells (right) in the spleens of WT and *Lrrk2*^−*/*−^ mice after pristane injection for 9 months. Each group had at least six mice for analysis. *p < 0.05

To explore the mechanisms of less production of total IgG and autoantibody in *Lrrk2*^−*/*−^ mice, we further analyzed B cell terminal differentiation closely related to antibody production after pristane injection. Germinal center (GC) B cells and plasma cells are two key subpopulations at the terminal stage of B cell differentiation occurring in GCs responsible for antibody production upon antigen stimulation [30]. At present, it is not clear how pristane induces the release of autoantigens and triggers B cell differentiation. Nevertheless, we were inclined to compare the percentages of GC B cells and plasma cells between WT and *Lrrk2*^−*/*−^ mice at 9 months post pristane treatment. Consistent with the antibody results, *Lrrk2*^−*/*−^ mice displayed the remarkable decrease in GC B cells and plasma cells in the spleens upon pristane treatment (Fig. 6a, b). Therefore, pristane-induced LRRK2 deficient mice exhibit impaired B cell terminal differentiation, which is probably responsible for the attenuated Ig production and subsequent less IC deposit in the kidneys.

## Discussion

SLE is an autoimmune disease that manifests the generation of autoantibodies and the deposition of ICs in certain tissues and organs. Autoreactive B cells are a key mediator for disease progression. While their hyperactivation is supposed to be exaggerated by type I interferon [31] or TLR7/9 signaling [32], B cell differentiation in SLE is also programmed by multiple factors [1, 33]. In this study, the role of LRRK2 as a novel potential regulator in the pathogenesis of SLE was suggested based on the correlation of abnormal LRRK2 expression in B cells with disease severity of SLE, and further validated in a pristane-induced lupus-prone model. The engagement of LRRK2 in the pathogenesis of SLE was probably through affecting B cell terminal differentiation and the production of pathological antibodies.

LRRK2 is a kinase protein with its variants associated with multiple diseases including Parkinson’s disease (PD) [12], IBD [15, 16] and cancer [17]. A recent report also suggested that LRRK2 variant was associated with SLE [9]. Interestingly, LRRK2 was previously reported to be highly expressed in B cells [21]. We extend this notion by showing abnormally high expression of LRRK2 in B cells from SLE patients. Furthermore, we demonstrated that abnormal expression of LRRK2 in B cells was correlated with SLEDAI, C3 and C4 levels which were key standards for SLE diagnosis. These data also suggest that LRRK2 may have a critical role in B cell immunity. Surprisingly, we couldn’t observe any effects of LRRK2 on B cell development and humoral immune response at the steady state in mice, which is inconsistent with a previous study [34]. The reason for this discrepancy is still unknown. One possible reason may be the environment difference which may lead to microbiota change. It’s worth to address this point in the future.

LRRK2 is highly expressed in B cells, monocytes, and dendritic cells suggesting its potential function in immune system [11]. LRRK2 expression is induced by interferon-γ (IFN-γ) and lipopolysaccharide (LPS) which is important for its antimicrobial activities [11]. Its deficiency exacerbated colitis in mice treated with dextran sodium sulphate [16]. Mechanistic studies showed that LRRK2 was a potent negative regulator of the transcription factor NFAT [16]. Recently, LRRK2 was demonstrated to be required for lysozyme sorting in Paneth cells directed by commensal bacteria through a Nod2–LRRK2–Rab2a axis [18]. We recently reported LRRK2 was critical for NLRC4 inflammasome activation [20]. Collectively, these emerging data implies the possible roles of LRRK2 in immune modulation. While many of these studies suggest that LRRK2 is critical in innate immunity, its role in adaptive immunity is largely unknown. Here we found that abnormal LRRK2 expression was closely associated with SLE severity. LRRK2 promoted B cell maturation and hyper-activation, increased autoantibody production and aggravated pristane-induced lupus in a SLE animal model, thus implicating LRRK2 as a new regulator of B cell function and humoral immunity. The underlying mechanisms warrant further investigation.

*LRRK2* gene polymorphism is originally dedicated to PD incidence, a disease characterized as a neurodegenerative disorder with the loss of midbrain dopaminergic neurons [13]. However, more and more reports describe the occurrence of inflammation and the elevation of inflammatory cytokines in the brains and cerebrospinal, alternatively supporting the roles of LRRK2 in immune regulation and making it a potential target for disease control. Several favorable candidate inhibitors have been developed for the experimental control of PD based on the kinase activity of LRRK2, such as LRRK2-IN-1 [35], CZC-54252 and CZC-25146 [36], GSK2578215A [37] etc. The LRRK2 inhibitor, PF-06447475, effectively attenuates α-synuclein-induced dopaminergic neurodegeneration [38]. In addition, LRRK2 inhibitor treatment appears to promote the ubiquitination of LRRK2 and consequently accelerate its proteasomal degradation [39]. Considering the increase of LRRK2 expression in B cells and the involvement in the pathogenesis of SLE, whether the existing LRRK2 inhibitor can specifically retard the B cell activation and differentiation for disease remission is worthy of further exploration.

## Conclusion

In summary, our study present here provides the direct evidence on the involvement of LRRK2 in the pathogenesis of SLE in mice. This is probably due to its roles in regulating B cell terminal differentiation, which in turn affects the antibody production and disease progression. Considering the exclusively high expression of LRRK2 in B cells from SLE patients, the pathological roles of LRRK2 in human SLE might be similar to a large extent. Once the underlying mechanisms are clarified, LRRK2 might become a novel target for developing new diagnostic and therapeutic strategy for the control of SLE.

## Additional file

**Additional file 1: Figure S1.** The purity of CD4^+^T cells and B cell. CD4^+^T cells and B cells from SLE patients or HCs were isolated by using a human CD4 MicroBeads kit and a human B Cell Enrichment Kit respectively. The purity of CD4^+^T cells and B cells were determined by flow cytometry. Cells with the purity > 95% were used for further experiments. **Figure S2.** LRRK2 expression in WT and *Lrrk2*^−/−^ mice. WT and *Lrrk2*^−/−^ mice splenocytes were prepared and protein expression levels of LRRK2 (top row) and hsp90 (bottom row) were analyzed by Western blot. **Figure S3.** Antibody dynamics in pristane-induced lupus-like mice. Sera were collected every 4 weeks from the mice. Antibody levels in the sera of WT and *Lrrk2*^−/−^ mice, including total IgG, total IgM, anti-SnRNP antibody and anti-dsDNA antibody were assayed by ELISA.

## Abbreviations

SLE: systemic lupus erythematosus
LRRK2: leucine-rich repeat kinase 2
SLEDAI: systemic lupus erythematosus disease activity index
pDCs: plasmacytoid dendritic cells
GWAS: genome-wide associated analysis
IBD: inflammatory bowel disease
PBMCs: peripheral blood mononuclear cells
CVID: common variable immunodeficiency
snRNPs: small nuclear ribonucleoproteins
GC: germinal center
PD: Parkinson’s disease

## References

[1] Tsokos GC (2011). Systemic lupus erythematosus. N Engl J Med.

[2] Dorner T, Giesecke C, Lipsky PE (2011). Mechanisms of B cell autoimmunity in SLE. Arthritis Res Ther.

[3] Beckwith H, Lightstone L (2014). Rituximab in systemic lupus erythematosus and lupus nephritis. Nephron Clin Pract.

[4] Martínez R, Muñoz A, Velloso ML, Rodríguez Montero S, Belmonte-López MA, Marenco JL (2010). Rituximab is effective in the treatment of nephritis in lupus patients, refractory to conventional immunosuppressive therapy. J Transl Med.

[5] Malkiel S, Barlev AN, Atisha-Fregoso Y, Suurmond J, Diamond B (2018). Plasma cell differentiation pathways in systemic lupus erythematosus. Front Immunol.

[6] Dolff S, Bijl M, Huitema MG, Limburg PC, Kallenberg CG, Abdulahad WH (2011). Disturbed Th1, Th2, Th17 and T(reg) balance in patients with systemic lupus erythematosus. Clin Immunol.

[7] Alculumbre S, Raieli S, Hoffmann C, Chelbi R, Danlos FX, Soumelis V. Plasmacytoid pre-dendritic cells (pDC): from molecular pathways to function and disease association. Semin Cell Dev Biol. 2018. 10.1016/j.semcdb.2018.02.014 29444460

[8] Cui Y, Sheng Y, Zhang X (2013). Genetic susceptibility to SLE: recent progress from GWAS. J Autoimmun.

[9] Zhang YM, Zhou XJ, Cheng FJ, Qi YY, Hou P, Zhao MH, Zhang H (2017). Autophagy-related gene LRRK2 is likely a susceptibility gene for systemic lupus erythematosus in northern Han Chinese. Oncotarget.

[10] Zimprich A, Biskup S, Leitner P, Lichtner P, Farrer M, Lincoln S, Kachergus J, Hulihan M, Uitti RJ, Calne DB (2004). Mutations in LRRK2 cause autosomal-dominant parkinsonism with pleomorphic pathology. Neuron.

[11] Gardet A, Benita Y, Li C, Sands BE, Ballester I, Stevens C, Korzenik JR, Rioux JD, Daly MJ, Xavier RJ, Podolsky DK (2010). LRRK2 is involved in the IFN-gamma response and host response to pathogens. J Immunol.

[12] Rideout HJ, Stefanis L (2014). The neurobiology of LRRK2 and its role in the pathogenesis of Parkinson’s disease. Neurochem Res.

[13] Juarez-Flores DL, Gonzalez-Casacuberta I, Ezquerra M, Bano M, Carmona-Pontaque F, Catalan-Garcia M, Guitart-Mampel M, Rivero JJ, Tobias E, Milisenda JC (2018). Exhaustion of mitochondrial and autophagic reserve may contribute to the development of LRRK2 (G2019S)-Parkinson’s disease. J Transl Med.

[14] Wang D, Xu L, Lv L, Su LY, Fan Y, Zhang DF, Bi R, Yu D, Zhang W, Li XA (2015). Association of the LRRK2 genetic polymorphisms with leprosy in Han Chinese from Southwest China. Genes Immun.

[15] Liu Z, Lenardo MJ (2012). The role of LRRK2 in inflammatory bowel disease. Cell Res.

[16] Liu Z, Lee J, Krummey S, Lu W, Cai H, Lenardo MJ (2011). The kinase LRRK2 is a regulator of the transcription factor NFAT that modulates the severity of inflammatory bowel disease. Nat Immunol.

[17] Ruiz-Martinez J, de la Riva P, Rodriguez-Oroz MC, Mondragon Rezola E, Bergareche A, Gorostidi A, Gago B, Estanga A, Larranaga N, Sarasqueta C (2014). Prevalence of cancer in Parkinson’s disease related to R1441G and G2019S mutations in LRRK2. Mov Disord.

[18] Zhang Q, Pan Y, Yan R, Zeng B, Wang H, Zhang X, Li W, Wei H, Liu Z (2015). Commensal bacteria direct selective cargo sorting to promote symbiosis. Nat Immunol.

[19] Toledo Pinto TG, Batista-Silva LR, Medeiros RCA, Lara FA, Moraes MO (2018). Type I interferons, autophagy and host metabolism in leprosy. Front Immunol.

[20] Liu W, Liu X, Li Y, Zhao J, Liu Z, Hu Z, Wang Y, Yao Y, Miller AW, Su B (2017). LRRK2 promotes the activation of NLRC4 inflammasome during Salmonella Typhimurium infection. J Exp Med.

[21] Maekawa T, Kubo M, Yokoyama I, Ohta E, Obata F (2010). Age-dependent and cell-population-restricted LRRK2 expression in normal mouse spleen. Biochem Biophys Res Commun.

[22] Warnatz K, Wehr C, Drager R, Schmidt S, Eibel H, Schlesier M, Peter HH (2002). Expansion of CD19(hi)CD21(lo/neg) B cells in common variable immunodeficiency (CVID) patients with autoimmune cytopenia. Immunobiology.

[23] Nicholas MW, Dooley MA, Hogan SL, Anolik J, Looney J, Sanz I, Clarke SH (2008). A novel subset of memory B cells is enriched in autoreactivity and correlates with adverse outcomes in SLE. Clin Immunol.

[24] Liu Z, Zeng W, Huang X, Wang S, Zheng J, Pan M, Wang Y (2017). Peripheral CD19(hi) B cells exhibit activated phenotype and functionality in promoting IgG and IgM production in human autoimmune diseases. Sci Rep.

[25] Chen M, Daha MR, Kallenberg CG (2010). The complement system in systemic autoimmune disease. J Autoimmun.

[26] Wang Y, Horvath O, Hamm-Baarke A, Richelme M, Gregoire C, Guinamard R, Horejsi V, Angelisova P, Spicka J, Schraven B (2005). Single and combined deletions of the NTAL/LAB and LAT adaptors minimally affect B-cell development and function. Mol Cell Biol.

[27] Lopes-Carvalho T, Kearney JF (2004). Development and selection of marginal zone B cells. Immunol Rev.

[28] Reeves WH, Lee PY, Weinstein JS, Satoh M, Lu L (2009). Induction of autoimmunity by pristane and other naturally occurring hydrocarbons. Trends Immunol.

[29] Garin EH, Donnelly WH, Shulman ST, Fernandez R, Finton C, Williams RL, Richard GA (1979). The significance of serial measurements of serum complement C3 and C4 components and DNA binding capacity in patients with lupus nephritis. Clin Nephrol.

[30] Shlomchik MJ, Weisel F (2012). Germinal center selection and the development of memory B and plasma cells. Immunol Rev.

[31] Niewold TB, Hua J, Lehman TJ, Harley JB, Crow MK (2007). High serum IFN-alpha activity is a heritable risk factor for systemic lupus erythematosus. Genes Immun.

[32] Santiago-Raber ML, Baudino L, Izui S (2009). Emerging roles of TLR7 and TLR9 in murine SLE. J Autoimmun.

[33] Min HK, Kim SM, Park JS, Byun JK, Lee J, Kwok SK, Park YW, Cho ML, Park SH (2016). Fn14-Fc suppresses germinal center formation and pathogenic B cells in a lupus mouse model via inhibition of the TWEAK/Fn14 pathway. J Transl Med.

[34] Kubo M, Nagashima R, Ohta E, Maekawa T, Isobe Y, Kurihara M, Eshima K, Iwabuchi K, Sasaoka T, Azuma S (2016). Leucine-rich repeat kinase 2 is a regulator of B cell function, affecting homeostasis, BCR signaling, IgA production, and TI antigen responses. J Neuroimmunol.

[35] Koshibu K, van Asperen J, Gerets H, Garcia-Ladona J, Lorthioir O, Courade JP (2015). Alternative to LRRK2-IN-1 for pharmacological studies of Parkinson’s disease. Pharmacology.

[36] Ramsden N, Perrin J, Ren Z, Lee BD, Zinn N, Dawson VL, Tam D, Bova M, Lang M, Drewes G (2011). Chemoproteomics-based design of potent LRRK2-selective lead compounds that attenuate Parkinson’s disease-related toxicity in human neurons. ACS Chem Biol.

[37] Reith AD, Bamborough P, Jandu K, Andreotti D, Mensah L, Dossang P, Choi HG, Deng X, Zhang J, Alessi DR, Gray NS (2012). GSK2578215A; a potent and highly selective 2-arylmethyloxy-5-substitutent-*N*-arylbenzamide LRRK2 kinase inhibitor. Bioorg Med Chem Lett.

[38] Daher JP, Abdelmotilib HA, Hu X, Volpicelli-Daley LA, Moehle MS, Fraser KB, Needle E, Chen Y, Steyn SJ, Galatsis P (2015). Leucine-rich repeat kinase 2 (LRRK2) pharmacological inhibition abates alpha-synuclein gene-induced neurodegeneration. J Biol Chem.

[39] Zhao J, Molitor TP, Langston JW, Nichols RJ (2015). LRRK2 dephosphorylation increases its ubiquitination. Biochem J.

