# Supplementary material for: LRRK2 is involved in the pathogenesis of system lupus erythematosus through promoting pathogenic antibody production
Source: J Transl Med. 2019 Jan 22;17:37. doi: 10.1186/s12967-019-1786-6 (PMC6343316; doi:10.1186/s12967-019-1786-6)
Supplement: Supplementary file 1 — Additional file 1: Figure S1. The purity of CD4+T cells and B cell. CD4+T cells and B cells from SLE patients or HCs were isolated by using a human CD4 MicroBeads kit and a human B Cell Enrichment Kit respectively. The purity of CD4+T cells and B cells were determined by flow cytometry. Cells with the purity > 95% were used for further experiments. Figure S2. LRRK2 expression in WT and Lrrk2−/− mice. WT and Lrrk2−/− mice splenocytes were prepared and protein expression levels of LRRK2 (top row) and hsp90 (bottom row) were analyzed by Western blot. Figure S3. Antibody dynamics in pristane-induced lupus-like mice. Sera were collected every 4 weeks from the mice. Antibody levels in the sera of WT and Lrrk2−/− mice, including total IgG, total IgM, anti-SnRNP antibody and anti-dsDNA antibody were assayed by ELISA. [file 12967_2019_1786_MOESM1_ESM.docx]

**Additional file 1**

**LRRK2 is involved in the pathogenesis of system lupus erythematosus through promoting pathogenic antibody production**

Meiyu Zhang^1#^, Chengcheng Yao^1#^, Jun Cai^6#^, Shuai Liu^1^, Xia-nan Liu^5^, Yingying Chen^1^, Shujun Wang^1^, Ping Ji^1^, Meng Pan^2^, Zizhen Kang^3,4^, Ying Wang^1^

#: contributed equally to this work

To whom it should correspond:

Dr. Ying Wang

1. Shanghai Institute of Immunology, Department of Immunology and Microbiology, Shanghai Jiao Tong University School of Medicine, Shanghai, 200025, China

and

Dr. Zizhen Kang

3. Department of Inflammation and Immunity, Cleveland Clinic, Cleveland, OH, 44195, USA


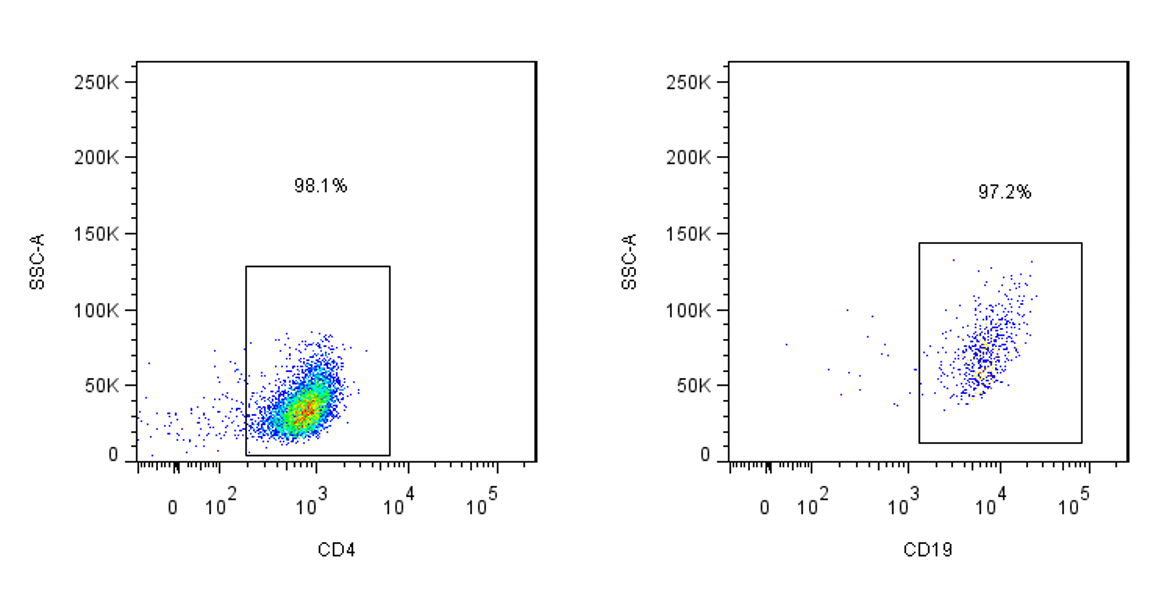
4. Department of Molecular Medicine, Cleveland Clinic Lerner College of Medicine, Case Western Reserve University, Cleveland, OH, 44106, USA

**Supplementary figure. 1. The purity of CD4^+^ T cells and B cell**

CD4^+^ T cells and B cells from SLE patients or HCs were isolated by using a human CD4 MicroBeads kit and a human B Cell Enrichment Kit respectively. The purity of CD4^+^ T cells and B cells were determined by flow cytometry. Cells with the purity > 95% were used for further experiments.


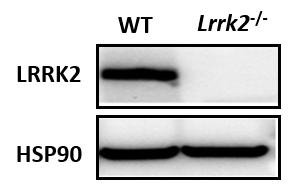


**Supplementary figure. 2. LRRK2 expression in WT and *Lrrk2^-/-^* mice**

WT and *Lrrk2^-/-^* mice splenocytes were prepared and protein expression levels of LRRK2 (top row) and hsp90 (bottom row) were analyzed by Western blot.


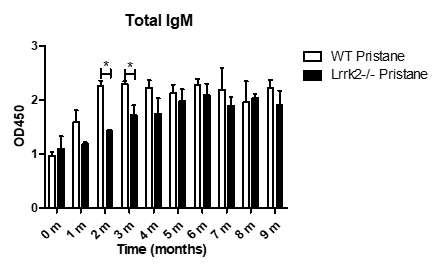

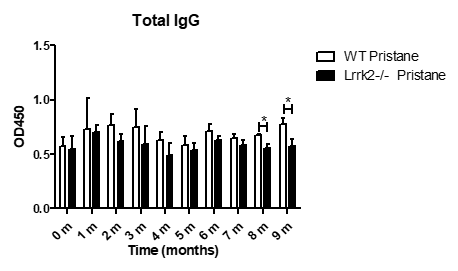


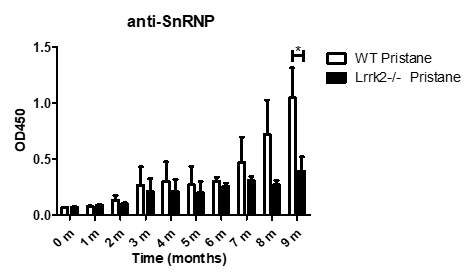


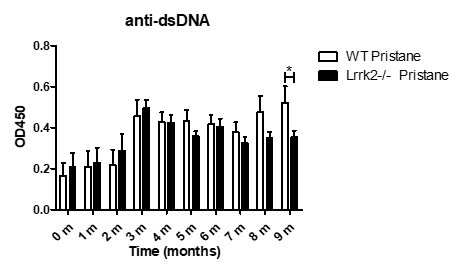


**Supplementary figure. 3. Antibody dynamics in pristane-induced lupus-like mice**

Sera were collected every 4 weeks from the mice. Antibody levels in the sera of WT and *Lrrk2^-/-^* mice, including total IgG, total IgM, anti-SnRNP antibody and anti-dsDNA antibody were assayed by ELISA.
